# Declining rates of health problems associated with crack smoking during the expansion of crack pipe distribution in Vancouver, Canada

**DOI:** 10.1186/s12889-017-4099-9

**Published:** 2017-02-03

**Authors:** Amy Prangnell, Huiru Dong, Patricia Daly, M. J. Milloy, Thomas Kerr, Kanna Hayashi

**Affiliations:** 1 British Columbia Centre for Excellence in HIV/AIDS, St. Paul’s Hospital, Urban Health Research Initiative, 608-1081 Burrard Street, Vancouver, BC V6Z 1Y6 Canada; 2 School of Population and Public Health, University of British Columbia, 206 E Mall, Vancouver, BC V6T 1Z9 Canada; 3 Vancouver Coastal Health, #800-601 West Broadway, Vancouver, BC V5Z 4C2 Canada; 4 Department of Medicine, University of British Columbia, St. Paul’s Hospital, 608-1081 Burrard Street, Vancouver, BC V6Z 1Y6 Canada; 5 Faculty of Health Sciences, Simon Fraser University, Blusson Hall, 8888 University Drive, Burnaby, BC V5A 1S6 Canada

**Keywords:** Crack smoking, Crack pipe acquisition, Harm reduction

## Abstract

**Background:**

Crack cocaine smoking is associated with an array of negative health consequences, including cuts and burns from unsafe pipes, and infectious diseases such as HIV. Despite the well-established and researched harm reduction programs for injection drug users, little is known regarding the potential for harm reduction programs targeting crack smoking to reduce health problems from crack smoking. In the wake of recent crack pipe distribution services expansion, we utilized data from long running cohort studies to estimate the impact of crack pipe distribution services on the rates of health problems associated with crack smoking in Vancouver, Canada.

**Methods:**

Data were derived from two prospective cohort studies of community-recruited people who inject drugs in Vancouver between December 2005 and November 2014. We employed multivariable generalized estimating equations to examine the relationship between crack pipe acquisition sources and self-reported health problems associated with crack smoking (e.g., cut fingers/sores, coughing blood) among people reported smoking crack.

**Results:**

Among 1718 eligible participants, proportions of those obtaining crack pipes only through health service points have significantly increased from 7.2% in 2005 to 62.3% in 2014 (*p* < 0.001), while the rates of reporting health problems associated with crack smoking have significantly declined (*p* < 0.001). In multivariable analysis, compared to those obtaining pipes only through other sources (e.g., on the street, self-made), those acquiring pipes through health service points only were significantly less likely to report health problems from smoking crack (adjusted odds ratio: 0.82; 95% confidence interval: 0.73–0.93).

**Conclusions:**

These findings suggest that the expansion of crack pipe distribution services has likely served to reduce health problems from smoking crack in this setting. They provide evidence supporting crack pipe distribution programs as a harm reduction service for crack smokers.

## Background

Crack cocaine use remains a significant public health problem in many parts of the world [1, 2]. A previous study documented that among 1936 persons who inject drugs surveyed across seven major cities in Canada, approximately 65.2% reported crack smoking in the last 6 months, and in Toronto 88.8% did so [3]. Further a significant increase in crack smoking has been shown among persons who inject drugs in Vancouver from 7.4% in 1996 to 42.6% in 2005 [4]. The negative consequences that can result from crack smoking range from extreme social marginalization to elevated morbidity and mortality [5, 6]. Of particular concern, users suffer from high rates of infectious diseases, such as HCV and HIV [1, 5]. Additionally, sores on the lips and mouth from smoking crack cocaine, which are common amongst users [7], provide a route for the transmission of infectious diseases when users do not have access to sterile and proper crack pipes and are compelled to share a pipe with others [8, 9]. Further exacerbating the risks of transmission and other health problems is the makeshift equipment used by crack smokers when no safe equipment is available, including wire scouring pads and glass stems, both of which have concerns of breaking and causing cuts [10]. Brillo screens, which are steel wool impregnated with soap, are also known to break apart during use, allowing for the particles to be inhaled and lead to breathing problems [11]. The use of unsafe smoking equipment, also contributes to the experience of pipes exploding while smoking, further contributing to the high reports of burns and lesions among users [12].

The Downtown Eastside (DTES) of Vancouver is home to Canada’s largest open drug scene [13], where a range of harm reduction programs and addiction treatments, including a supervised injection facility, also exist [14]. Beginning in 2011, in response to escalating crack smoking and resulting health concerns [15], the local health authority, Vancouver Coastal Health, implemented a Safer Smoking Pilot Project [15], which provided sterile crack cocaine smoking paraphernalia at no cost. Through the participation of community health programs and services, over 100,000 safer smoking kits were distributed to users from December 2011 to November 2012, through the coordination at over 7 distribution sites. After the initial pilot study ended, the distribution of crack pipes continued as a harm reduction program in the community.

While there is substantial evidence indicating that harm reduction strategies, including supervised injection sites and needle exchange programs [16–18], are effective in reducing the harms and improving the lives of people who inject drugs [19], there is a dearth of research examining the impact of crack pipe distribution programs among non-injecting users of crack. Drawing data from long-running prospective cohorts of people who use drugs in Vancouver, we sought to determine if the increased availability of safe crack smoking equipment through various health service points was associated with a decrease of health problems related to crack smoking in this setting.

## Methods

### Study procedures

The Vancouver Injection Drug Users Study (VIDUS) and the AIDS Care Cohort to evaluate Exposure to Survival Services (ACCESS) are ongoing open prospective cohorts of adult drug users recruited through word of mouth, street outreach, and referrals from community organizations in Vancouver, Canada. These studies have been described in detail previously [20]. Briefly, VIDUS enrolls HIV-negative persons who reported injecting an illicit drug at least once in the month preceding enrollment; ACCESS enrolls HIV-positive individuals who report using an illicit drug (other than, or in addition to, cannabis) in the previous month. For both cohorts, other eligibility criteria included being aged 18 years or older, residing in the greater Vancouver region and providing written informed consent. The study instruments and all other follow-up procedures for each study are essentially identical to allow for combined analyses. At baseline and semi-annually thereafter, participants complete an interviewer-administered questionnaire eliciting sociodemographic data as well as information pertaining to drug use patterns, risk behaviors, and health care utilization. Nurses collect blood samples for HIV and hepatitis C virus serology, provide basic medical care and arrange referrals to appropriate health care services if required. Participants receive a $30 (CDN) honorarium for each study visit. The University of British Columbia/Providence Healthcare Research Ethics Board provided ethical approval for both studies.

All participants who were enrolled in the cohorts between December 1, 2005 (the start date of the VIDUS and ACCESS cohorts) and November 30, 2014 (the most recent follow-up period available for the present analysis), and who reported ever injecting drugs preceding the baseline interview were included in the present analysis. Additionally, at each follow up, the sample was restricted to individuals who reported smoking crack cocaine in the previous 6 months because the analysis was focused on crack cocaine smoking.

### Study variables

The primary outcome of interest was experiencing health problems associated with smoking crack in the previous 6 months. As in a previous study [21], this was defined as reporting at least one of the following health problems: “Burns”, “Mouth sores”, “Cut fingers / sores”, “Raw throat”, or “Coughing blood” to the question within the interviewer administered questionnaire:: “In the past 6 months, have you experienced any of the following health problems from smoking crack?”

The primary explanatory variable of interest was crack pipe acquisition source in the previous 6 months. This was defined as reporting health service points only (e.g. needle exchange programs, health clinics, temporary shelters) *vs.* a mix of health service points and other sources *vs.* other sources only (e.g. street, homemade, corner store), to the question: “In the past 6 months, where did you get your crack pipes?”

We also considered secondary explanatory variables that might confound the relationship between crack pipe acquisition sources and reporting health problems from smoking crack. These included sociodemographic characteristics, including: age (per year older); biological sex at birth (female *vs.* male); ancestry (white *vs.* non-white); residing in the DTES in the previous 6 months (yes *vs.* no); homelessness in the previous 6 months, defined as having no fixed address, sleeping on the street, or staying in a shelter or hostel (yes *vs.* no); involvement in drug dealing in the previous 6 months (yes *vs.* no); involvement in sex work in the previous 6 months (yes *vs.* no); educational attainment (less than high school *vs.* high school completion or higher). Drug-use variables referred to behaviours in the previous 6 months, and included: ≥ daily crack smoking (yes *vs.* no); ≥ daily non-injection crystal methamphetamine use (yes *vs.* no); binge non-injection drug use, defined as compulsive high-intensity non-injection drug use that exceeds normal patterns of consumption (yes *vs.* no) [22]; shared crack pipe (yes *vs.* no); and rushed crack smoking while in public (yes *vs.* no). Other exposures and health status included: being a victim of violence, defined as having been attacked, assaulted, or suffered violence in the previous 6 months (yes *vs.* no); being HIV infected (yes *vs.* no); and incarceration in the previous 6 months (yes *vs.* no). All variable definitions are consistent with previous studies [23–25].

### Statistical analysis

As a first step, we examined the baseline sample characteristics stratified by reports of experiencing health problems from smoking crack, using the Pearson’s Chi-squared test (for binary variables) and Wilcoxon Rank Sum test (for continuous variables). Fisher’s exact test was used when one or more of the cells contained expected values less than or equal to five. First, we examined the temporal trends of crack pipe acquisition source and health problems, respectively, using univariable GEE models including the calendar dates of 6-month follow-up periods (per period later) as the independent variable.

Since the analyses of experiencing health problems included serial measures for each participant, we used generalized estimating equations (GEE) with logit link, which provided standard errors adjusted by multiple observations per person using an exchangeable correlation structure. We first used bivariable GEE analyses to examine the association between each explanatory variable and experiencing health problems associated with smoking crack. To examine the relationship between crack pipe acquisition source and health problems, we fit multivariable GEE models using a conservative confounding model selection approach [26]. We included all variables that were associated with reporting health problems in unadjusted analyses at *p* < 0.10 in a full multivariable model, and used a stepwise approach to fit a series of reduced models. After comparing the value of the coefficient of the crack pipe acquisition source in each reduced model, we dropped the secondary variable associated with the smallest relative change. We continued this iterative process until the minimum change exceeded 5%. In order to examine if the estimates differed for women and men, we have also repeated the model using an interaction term for the primary explanatory variable and sex. In order to examine whether the attrition towards the end of the study period biased the estimates, we also conducted a sensitivity analysis where we repeated the analyses among those whose last study visit was earlier than December 2013 (i.e., 1 year before the end of the study period). All *p*-values are two sided. All statistical analyses were performed using SAS software version 9.4 (SAS, Cary, NC).

## Results

In total, 1718 participants were eligible for the present study. Among this sample, 602 (35.0%) were women, 1018 (59.3%) self-reported white ancestry and the median age at baseline was 41.8 years (interquartile range [IQR] = 35.4–47.8). Overall, the 1718 individuals contributed 11,034 observations to the analysis and the median number of follow-up visits was 5 (IQR: 2–10) per person. The baseline characteristics of all participants stratified by reporting health problems associated with crack smoking are presented in Table 1.

**Table 1:** Baseline sample characteristics, stratified by reporting health problems associated with crack smoking in the past 6 months among crack smokers in Vancouver, Canada (*n* = 1718)

Characteristic	Experienced crack related health problems^a^		Odds Ratio (95% CI)	*p-*value
	Yes n (%) 587 (34.2)	No n (%) 1131 (65.8)		
Crack pipe acquisition source				
Health service points only	74 (12.6)	167 (14.8)	0.86 (0.64–1.16)	0.318
A mix of health service points and other sources	58 (9.9)	81 (7.1)	1.39 (0.97–1.98)	0.070
Other sources only	455 (77.5)	883 (78.1)		
Female sex	235 (40.0)	367 (32.4)	1.39 (1.13–1.71)	0.002
Age (median, IQR)	41 (34–47)	42 (36–48)	0.99 (0.97–1.00)	0.017
Caucasian	334 (56.9)	684 (60.5)	0.86 (0.70–1.06)	0.152
Completed < high school	289 (49.2)	578 (51.1)	0.93 (0.76–1.13)	0.456
DTES residency^a^	438 (74.6)	809 (71.5)	1.17 (0.93–1.47)	0.174
Homeless^a^	229 (39.0)	413 (36.5)	1.11 (0.91–1.37)	0.305
≥ Daily crack smoking^a^	343 (58.4)	458 (40.5)	2.06 (1.68–2.53)	<0.001
≥ Daily non-injection meth use^a^	4 (0.7)	17 (1.5)	0.45 (0.15–1.34)	0.142
Binge non-injection drug use^a^	225 (38.3)	290 (25.6)	1.80 (1.46–2.23)	<0.001
Shared crack pipe^a^	473 (80.6)	719 (63.6)	2.37 (1.87–3.01)	<0.001
Rushed public crack smoking^a^	199 (33.9)	281 (24.8)	1.57 (1.26–1.95)	<0.001
Drug dealing^a^	255 (43.4)	368 (32.5)	1.59 (1.30–1.96)	<0.001
Sex work^a^	122 (20.8)	160 (14.1)	1.61 (1.24–2.09)	<0.001
A victim of violence^a^	188 (32.0)	218 (19.3)	1.99 (1.58–2.50)	<0.001
Incarceration^a^	124 (21.1)	182 (16.1)	1.40 (1.09–1.81)	0.009
HIV positive	240 (40.9)	458 (40.5)	1.02 (0.83–1.24)	0.876

*PWID* People who inject drugs, *CI* confidence interval, *IQR* interquartile range

*DTES* Downtown Eastside

^a^ Denotes activities in the previous 6 months

**Fig. 1:**
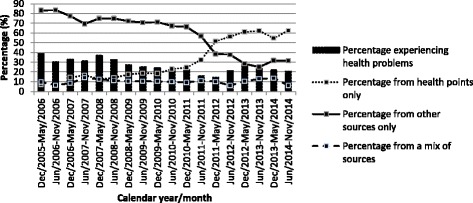
Percentages of reporting health problems associated with crack smoking and crack pipe acquisition sources

As shown in Fig. 1, the proportion reporting health problems declined from 39.2% at baseline (December 2005 – May 2006) to 20.7% during the last follow-up period (June 2014 – November 2014), and the declining trend was statistically significant (*p* < 0.001). Additionally, the proportion of those obtaining crack pipes only through health service points increased significantly from 7.2% in 2005 to 62.3% in 2014, while the rates obtaining from other sources only decreased significantly from 83.2% in 2005 to 31.5% in 2014 (*p* < 0.001). Figure 1 depicts the increase in obtaining crack pipes from health service points only, beginning in approximately 2011 which coincides with the implementation of the safer crack pipe smoking distribution program by the local health authority as described above.

The results of the bivariable and multivariable GEE analyses of reporting health problems associated with crack smoking are presented in Table 2. As shown, in the final multivariable model after adjusting for a range of potential confounders, obtaining crack pipes through health service points remained significantly and negatively associated with reporting health problems (adjusted odds ratio [AOR] = 0.82; 95% confidence interval [CI]: 0.73–0.93), while obtaining pipes through a mix of health service points and other sources was only marginally associated (AOR = 1.17; 95% CI: 1.00–1.36). When we repeated the multivariable analysis using the interaction term between sex and crack pipe acquisition source, the results were not statistically different between women and men (*p*-value of the interaction term =0.460).

**Table 2:** Bivariable and multivariable GEE analyses of reporting health problems associated with crack smoking among crack smokers in Vancouver, Canada (*n* = 1718)

Characteristic	Unadjusted		Adjusted	
	Odds Ratio (95% CI)	*p-*value	Odds Ratio (95% CI)	*p-*value
Source				
Health service point only *vs.* Other sources only	0.70 (0.63–0.79)	<0.001	0.82 (0.73–0.93)	<0.001
A mix of health source points and other sources *vs.* Other sources only	1.23 (1.06–1.43)	0.006	1.17 (1.00–1.36)	0.051
Age				
(per year older)	0.97 (0.96–0.98)	<0.001	0.99 (0.98–0.99)	0.002
Sex				
(female *vs.* male)	1.37 (1.20–1.56)	<0.001	1.31 (1.15–1.50)	<0.001
Ethnicity				
(Caucasian *vs.* other)	1.00 (0.88–1.14)	0.994		
Less than high school diploma achieved				
(yes *vs.* no)	0.99 (0.87–1.13)	0.923		
DTES residency^a^				
(yes *vs.* no)	1.24 (1.11–1.40)	<0.001	1.09 (0.96–1.22)	0.178
Homelessness^a^				
(yes *vs.* no)	1.34 (1.22–1.48)	<0.001		
Daily non-injection crack smoking^a^				
(yes *vs.* no)	1.68 (1.53–1.84)	<0.001	1.29 (1.16–1.42)	<0.001
Daily non-injection meth use^a^				
(yes *vs.* no)	1.14 (0.72–1.81)	0.582		
Binge non-injection drug use^a^				
(yes *vs.* no)	1.64 (1.51–1.79)	<0.001	1.53 (1.40–1.67)	<0.001
Shared crack pipe ^a^				
(yes *vs.* no)	2.07 (1.88–2.28)	<0.001	1.73 (1.56–1.91)	<0.001
Rushed public crack smoking^a^				
(yes *vs.* no)	1.86 (1.66–2.08)	<0.001		
Drug dealing^a^				
(yes *vs.* no)	1.66 (1.50–1.83)	<0.001	1.25 (1.12–1.39)	<0.001
Sex work^a^				
(yes *vs.* no)	1.95 (1.70–2.23)	<0.001		
A victim of violence^a^				
(yes *vs.* no)	1.69 (1.52–1.88)	<0.001	1.47 (1.31–1.64)	<0.001
Incarceration^a^				
(yes *vs.* no)	1.56 (1.37–1.77)	<0.001		
HIV positive				
(yes *vs.* no)	1.06 (0.93–1.21)	0.359		

*GEE* generalized estimating equations, *PWID* People who inject drugs, *CI* confidence interval, *DTES* Downtown Eastside

^a^ Denotes activities in the previous 6 months

The sensitivity analysis included 431 participants whose last study visit was earlier than December 2013. The results were essentially the same as those of the primary analyses. In the simple GEE analyses, the declining trend for reporting health problems and the increasing trend for acquiring pipes through health service points only were both significant at *p* < 0.001. In the multivariable GEE analysis, obtaining crack pipes through health service points remained significantly and negatively associated with reporting health problems (AOR = 0.74; 95% CI: 0.55–0.99), while obtaining pipes through a mix of health service points and other sources was not (AOR = 0.85; 95% CI: 0.59–1.24).

## Discussion

We observed that the increase in crack pipe distribution services coincided with a corresponding increase in the uptake of crack pipes obtained through health service points only. Further, rates of reporting health problems associated with crack smoking declined significantly after the crack pipe distribution program was implemented. In the multivariable analysis, compared to obtaining crack pipes through other non-health service sources only, obtaining pipes through health service points only was significantly and negatively associated with reporting health problems from smoking crack. These findings suggest that the recent expansion of crack pipe distributions in this setting has likely served to reduce health problems experienced by crack smokers, achieving the desired outcome of the program.

While crack users are obtaining their safe crack smoking equipment from health service points, they may also be exposed to education around safer smoking techniques and practices, by being in direct contact with service providers in the community. This may also have the benefit of exposing drug users with no connections to health care to available providers in their area [27]. A previous study of an outreach-based crack smoking kit distribution service indicated that unsafe smoking practices such as using Brillo pads and sharing crack paraphernalia remained prevalent, even after the implementation of the service [10], suggesting the importance of placing such service in a continuum of broader health service system and ensuring the availability of smoking kits to reduce risky smoking behaviours.

Our findings of a reduction of health problems, are consistent with harm reduction programs for people who inject drugs [19], including needle exchange programs and supervised injection sites, where they are effective in reducing overall negative health consequences. By providing users with high-quality smoking equipment and reducing the dependence on unsafe equipment, the unintended negative consequences, including exploding pipes, burns, and inhaling brillo fragments, are further reduced.

This study has several limitations. First, the VIDUS and ACCESS cohorts are not random samples and therefore generalizability of the findings may be limited. Second, data used in the study, including those for the primary explanatory and outcome variables, were solely based on self-report and thus could be subject to reporting bias, including socially desirable responses. Although efforts were made to prompt participants to report all sources of crack pipes in the past 6 months, including opportunistic sources, the pipe sources may have been incorrectly categorized due to self-report bias. However, self-reported behavioural data has been shown to be largely accurate among adult drug-using populations [28]. Lastly, as with any observational research, unmeasured confounders may exist although we sought to reduce this bias through adjustment of statistical models using key predictors of health problems associated with crack smoking. As this was an observational study we cannot infer causation between crack pipe acquisition and experiencing health problems. Also, while we conducted the sensitivity analysis for participants who were lost to follow-up in one or more years prior to the end of the study period, and showed that the results remained the same, it is impossible to confirm whether attrition was random or not, and therefore there is still a possibility that attrition may have under- or over-estimated the results.

## Conclusion

In summary, our findings demonstrate that the uptake of crack pipes through health service points increased significantly during a period of expansion of crack pipe distribution, while the prevalence of health problems from smoking crack declined significantly during the same time period. Further, compared to obtaining pipes only through other sources (e.g., on the street, self-made), acquiring pipes through health service points only was significantly and negatively associated with reporting health problems from smoking crack. While we cannot infer causation from this observational study, these findings provide support for the distribution of safe crack smoking kits as an effective harm reduction measure for crack smokers. For communities experiencing high rates of crack cocaine smoking and the associated health problems, increased safe crack smoking equipment may serve to reduce health problems and conserve health care spending.

## Abbreviations

DTES: Downtown Eastside
HIV: Human immunodeficiency virus
